# What is “Hyper” in the ALS Hypermetabolism?

**DOI:** 10.1155/2017/7821672

**Published:** 2017-09-07

**Authors:** Alberto Ferri, Roberto Coccurello

**Affiliations:** ^1^Institute of Cell Biology and Neurobiology (IBCN), National Research Council (CNR), Via del Fosso di Fiorano 64, 00143 Rome, Italy; ^2^IRCCS Santa Lucia Foundation, Via del Fosso di Fiorano 64, 00143 Rome, Italy

## Abstract

The progressive and fatal loss of upper (brain) and lower (spinal cord) motor neurons and muscle denervation concisely condenses the clinical picture of amyotrophic lateral sclerosis (ALS). Despite the multiple mechanisms believed to underlie the selective loss of motor neurons, ALS aetiology remains elusive and obscure. Likewise, there is also a cluster of alterations in ALS patients in which muscle wasting, body weight loss, eating dysfunction, and abnormal energy dissipation coexist. Defective energy metabolism characterizes the ALS progression, and such paradox of energy balance stands as a challenge for the understanding of ALS pathogenesis. The hypermetabolism in ALS will be examined from tissue-specific energy imbalance (e.g., skeletal muscle) to major energetic pathways (e.g., AMP-activated protein kinase) and whole-body energy alterations including glucose and lipid metabolism, nutrition, and potential involvement of interorgan communication. From the point of view here expressed, the hypermetabolism in ALS should be evaluated as a magnifying glass through which looking at the ALS pathogenesis is from a different perspective in which defective metabolism can disclose novel mechanistic interpretations and lines of intervention.

## 1. Introduction

We may just portray amyotrophic lateral sclerosis (ALS) as a progressive and rapid degeneration of upper and lower motor neurons of the spinal cord, brainstem, and cerebral cortex. This precise, though laconic, clinical picture instantly seizes our attention on the gradual loss of motor function that ends with skeletal muscle atrophy, paralysis, and death. From an aetiological point of view, ALS is an inexplicable multifactorial, multigenic, and multiorgan disease [1–3], for which no cure exists. Although the list of possible underlying pathogenetic mechanisms is constantly growing to include oxidative/nitrosative stress, protein misfolding/aggregation, defective autophagy, mitochondrial impairment, and excitotoxicity [4–7], a clear aetiology is still elusive. In most patients, ALS occurs in a sporadic form (sALS), while only 10% of ALS cases are inherited (familial ALS (fALS)). The two forms, sporadic and familial, display very similar clinical features and, despite the heterogeneity in symptoms, age at onset and disease duration, and the two forms are clinically indistinguishable [8]. In the last decade, different mutated genes have been discovered in familial cases of ALS and, in addition to the well-known mutations of *Sod1* gene, multiple animal models were generated to mimic the disease. In this view, since fALS and sALS are clinically overlapping, the genetic models might help to shed light on the more frequent sporadic form of the disease.

## 2. Muscle Atrophy and Energy Consumption: The Paradox Wherein Wasting and Hypermetabolism Cohabitate

Considering the clinical onset of the disease involving spinal and bulbar motor neurons, patients with ALS inevitably will show dysphagia, mastication and respiratory difficulty, a major decrease in food intake, and energy deficit. Premorbid body mass index appears to increase the risk of ALS [9], and survival prognosis is less favourable in the presence of weight loss. Thus, patients experience weight loss during the progression of disease as a result of the decline of skeletal muscle mass and malnutrition-associated drop of fat and fat-free mass and lower body mass index [10–12]. There is an increasing number of evidence reporting the severe clinical consequences of lower motor neurons degeneration and associated alteration of eating habits and malnutrition in these patients. Moreover, upper limb weakness and reduced dexterity undermines the ability to keep patients' usual eating. Malnutrition is then further aggravated by the loss of appetite that represents a multifactorial component of the disease, reflecting the growing difficulties of eating and ALS-associated depressive symptoms.

Thus, underfeeding reduces energy intake further depleting energy stores and skeletal muscle contractile capacity, exacerbating weakness and loss of motor skills. In this view, ALS is a puzzling disease in which we recognize a vicious (and paradoxical) pathological circle involving atrophy of the highest energy-consuming tissue of the body and reduced physical activity along with concomitant increase of energy expenditure. The coexistence of two opponent processes is a challenge for disease understanding and a potential route to pursue for developing novel therapeutics. The enigmatic cooccurrence of insufficient energy intake and energy dissipation is the purpose of the present examination. Are we facing a deceitful problem or a better understanding of hypermetabolism can be a promising strategy to look at ALS?

## 3. Defective Energy Metabolism in ALS

Like ALS aetiology, the origin of defective energy homeostasis in ALS patients is currently obscure. The idea that defective energy metabolism can have a role in ALS pathogenesis requires to reexamine the contribution of skeletal muscle to ALS aetiology and symptomatology. Indeed, several evidence support an early occurrence of neuromuscular symptoms (e.g., atrophy, cachexia, and fasciculation) before the loss of motor neurons and neurodegeneration [13, 14]. In particular, fast-twitch muscle fibers are reported as more vulnerable than slow-twitch muscle in different studies on ALS murine models [14–16]. Apart from the controversy about the preeminence of the so-called “dying-back hypothesis,” the role of skeletal muscle as copartner in the upper and lower motoneuron degeneration is today acknowledged.

In spite of the reduction of fat-free mass, different studies have reported that familial and sporadic ALS patients have increased energy expenditure, particularly at rest [11, 17–22]. This is in clear conflict with the effects of undernutrition on the regulation of energy balance and defensive mechanisms aimed at lowering energy wastage [23]. A similar metabolic picture has been described also in transgenic mice line such as superoxide dismutase 1 (SOD1) G93A and G86R [24]. Here, it was showed that energy expenditure was increased in these transgenic ALS mice models, thus confirming the notion of defective energy metabolism as observed in ALS patients. A variety of different mechanisms have been hypothesized, but malfunctioning of energy processes such as oxidative stress and alteration of axonal transport and mitochondrial dysfunction are considered the most recurrent aspects in the study of ALS aetiology.

Although of unknown origin, excessive energy expenditure has been hypothesized to be dependent on defective mitochondrial function in liver, muscle, and spinal motoneurons [25, 26]. Indeed, abnormal mitochondrial morphology, localization, and transport have been described in motor neurons and muscles of ALS patients. Furthermore, a wide body of data reported alterations in mitochondrial functionality in ALS patients in several cellular populations such as neurons, glial cells, and muscle fibers [27]. Mitochondrial alterations such as fragmentation of the mitochondrial network, mitochondrial aggregation and accumulation, and abnormal mitochondrial dynamics are considered key factors in ALS pathogenesis that can occur in SOD1 G93A mice before disease onset and muscle paralysis [28].

Since metabolically active muscle mass is decreased in ALS, the skeletal muscle appears a possible first target of mitochondrial dysfunctions [29–32]. Indeed, a pioneer study reported the presence of aggregates of mitochondria in the subsarcolemmal region of skeletal muscles in ALS patients [33, 34], while increased mitochondria volume and morphological alterations were reported in muscle biopsies in ALS [35, 36]. Mitochondrial metabolic dysfunction such as decreased oxidative capacity and alteration in Ca^2+^ handling have been described in skeletal muscle of ALS animal models and patients [36]. Indeed, mitochondrial Ca^2+^ levels were found altered in muscle biopsy samples from patients with sporadic ALS [37]. Furthermore, in muscles from SOD1, G93A mice have been identified defects in mitochondrial structure and function associated with hyperactive Ca^2+^ release from the sarcoplasmic reticulum at neuromuscular junction regions prior to the onset of overt disease [38]. Alterations in mitochondrial complex activities were found decreased once again both in patients and in ALS animal models. Specifically, complex IV activity was found decreased in skeletal muscle from ALS patients [29] and the inhibition of complex I and II in hind- and forelimb muscles are key molecular signatures during early- and late-stage disease progression in the SOD1 G93A mouse model [39].

Mutant SOD1 proteins have been found localized in the mitochondria outer membrane and matrix and at the level of mitochondrial intermembrane space [40–42]. The linkage between SOD1 and mitochondria can for instance help to explain defective respiratory complex function, disease-associated increase of SOD1 activity in muscle, and decreased oxygen consumption in slow oxidative muscle fibers (i.e., soleus) but not in glycolytic fibers (i.e., EDL) from SOD1 G93A mice [43]. Reduced activity of mitochondrial complex I of the electron transport chain and cytochrome C oxidase in muscle, in spinal cord, and motor cortex has been frequently reported in patients and, as an early event, also in mutant SOD1 mice [44–46]. Although the mitochondrial localization of mutant SOD1 has been discussed by Bergemalm and colleagues [47] by considering the amount of mitochondrial mutant SOD1 in murine ALS models as a potential artifact due to the high copy numbers of the transgene, the role of mitochondrial SOD1 in ALS aetiology has been established in different non-SOD1 ALS models [48].

In the light of extensive experimental evidence, it is possible to conclude that mitochondrial dysfunction in ALS is not restricted to motor neurons and it appears clearly that the oxidative alterations observed in skeletal muscles are relevant to the progression of the disease. To further support this hypothesis, the overexpression of uncoupling protein-3 (UCP3) has been found in muscle biopsies from ALS patients and in nondenervated skeletal muscle but not spinal cord of SOD1 G86R mice, along with muscle-restricted depletion of ATP levels [49]. Finally, muscle-restricted overexpression of uncoupling protein 1 (UCP-1) leads to age-associated neurodegeneration of neuromuscular junction (NMJ) worsening motor neuron disease in SOD1 mice [50]. It is, however, worth mentioning that the mitochondrial damage can be absent in muscle fibers, as observed in a cohort of sporadic ALS patients [51].

## 4. The Bioenergetic Engine: Muscle Metabolic Properties and Hypermetabolism in ALS

The dispute around the *primum movens* (i.e., “who came first?”) in ALS pathophysiology is definitely misleading. Early events deranging muscle physiology prior denervation process and death of motor neuron units have been repeatedly described [13, 52], thus demonstrating the importance of muscle-to-motor dying back process. Given the multifactorial aetiology of ALS, it is possible that the understanding of alterations in skeletal muscle machinery could help to disclose the contribution of defective energy metabolism to the pathogenesis of motor neuron disease. Emblematically muscle-restricted expression of SOD1 genes induced motor neuron degeneration, muscle atrophy, oxidative damage, and mitochondrial dysfunction [52]. A further support for a relevant role of skeletal muscle alterations in ALS comes from a study by Dobrowolny and colleagues that showed the strong protective effect exerted by muscle-restricted expression of local IGF1 on SOD1 G93A mice [53]. IGF1 muscle-restricted expression protects NMJ motor, inhibits neuronal loss, and slows down disease progression. It is, however, appropriate to mention that the downregulation of mutant SOD1 expression, through siRNA, restricted in skeletal muscle of SOD1 G93A mice did not improve muscle strength, and muscular selective knockdown of the mutant *Sod1* transgene through Cre-Lox recombination did not affect disease progression of SOD1 G37R ALS mouse model [54]. Nevertheless, it is worth underlining that the decrease of expression obtained in muscles of mutant SOD1 was only 25%, evidence that, once again, corroborate the multifactorial (and elusive) nature of the pathology.

Muscle metabolism determines the major part of the whole-body energy metabolism, and the heterogeneous composition and the adaptive responses of the skeletal muscle machinery are shaped both by fiber type and intensity and duration of muscle exercise [55]. It is known that, depending on which type of myosin heavy chain (MHC) isoform, different adaptive responses to energy requests are possible. Hence, type I MHC is a slow-twitch fiber capable of higher oxidative volume with greater mitochondrial content and resistance to fatigue (i.e., endurance exercise). On the other hand, type IIb MHC is a low fatigue-resistant fast-twitch fiber that uses glucose and phosphocreatine as major fuel and for that is regarded as glycolytic. Somehow, intermediate between type I and type IIb are the type IIa fibers (mostly oxidative and fast contractile in mouse) [56]. From the different metabolic profile of muscle fibers stems immediately the type of movement they are best suited for. Indeed, prolonged muscle effort requires high oxidative energy sparing type I fibers, whereas brief and explosive movements require anaerobic high glycolytic and energy-consuming fibers. However, muscle fibers are dynamic entities that are primarily shaped and reshaped by external forces (i.e., movements). High-intensity or high-volume exercises are powerful forces capable of remodelling in opposite ways the muscle metabolic profile and promote for instance the switching between glycolytic and oxidative fiber type to meet the sustained energy request.

The time-course of disease development in ALS can be also described considering the type of muscle fibers early affected in the die-back process of motor neuron degeneration, which appears to preferentially involve the functional unit characterized by large motor axon/IIb muscle fiber [57, 58]. Selective functional decline and decrease of proportion of fast-twitch type IIb fibers is detectable as early as 60 days of age in the fast-twitch tibialis anterior (TA) of SOD1 mice [59]. Fiber-type transition from fast-fatigable fibers to fast intermediate and fatigue-resistant muscle fibers has been described in SOD1 mice [58, 59] together with a reduction of age-associated decrease of glycolytic fibers induced by the increase of neuromuscular activity [60]. In SOD1 mice at 130 days of age, the muscle phenotype of the extensor digitorum longus (EDL) was found switched from glycolytic to high oxidative and smaller-than-normal fatigue-resistant fibers [61].

Accordingly, SOD1 mice can display a greater endurance effort along with a reduced ability to use glucose as prevalent fuel in glycolytic fibers [62]. Remarkably, this study shows that in SOD1 mice, fast-twitch type IIb fibers underwent a drastic and early (65 days of age) metabolic switch from preferential glucose utilization to lipid metabolism occurring before the appearance of any measurable motor symptom. As consequence, SOD1 mice in presymptomatic stage also show a higher aerobic capacity and endurance ability that parallels the decrease of activity of phosphofructokinase 1 (PFK1), the rate-limiting enzyme of glycolysis, and therefore defective glucose utilization in skeletal muscle (i.e., glycogen accumulation) and inhibition of glycolytic pathway [62]. Moreover, this study shows an increased consumption of fatty acids and lipid-associated molecules in glycolytic muscle as well as the possibility to delay symptom onset by inhibiting the activity of pyruvate dehydrogenase kinase 4, thus stimulating glucose oxidation.

Interestingly, muscle fiber subtypes are differently affected in different pathologic conditions. Fiber damage, denervation-induced atrophy, and muscle immobilization preferentially affects type I fibers [63] while type II fibers appear more susceptible to cancer-induced cachexia, diabetes, and ageing [64]. The type II fiber-selective alteration in ALS mouse model appears to recapitulate age-associated decline in muscle mass (e.g., sarcopenia) and mimic the condition of nutrient-associated atrophy as observed in cancer (e.g., cachexia). From this point of view, the derangement of type II glycolytic fibers in SOD1 mice seems to mimic a persistent state of starvation and nutrient depletion. Considering the major contribution of skeletal muscle in glucose disposal, the dysfunction of fast-twitch type IIb fibers in murine model of ALS can dramatically affect energy substrate utilization. Skeletal muscle adapts very efficiently to changing nutrient availability by the alteration of substrate utilization and switching from lipid oxidation (in fasting conditions) to insulin-stimulated glucose consumption. Indeed, during fasting or prolonged exercise, skeletal muscles are required to adapt to either reduced energy intake or increased energy expenditure. Thus, whole body energy homeostasis reflects muscle metabolic flexibility and adaptive responses to changing substrate availability. Considering the contribution of skeletal muscle to energy metabolism, the impairment of glycolytic fibers observed in ALS mice models may be associated with metabolic disorders including insulin resistance and glucose intolerance that have been reported in ALS patients [65, 66]. Moreover, metabolic flexibility and ability of skeletal muscle to shift between glucose and lipid oxidation is reduced in insulin-resistant patients and subjects with type II diabetes [67]. To cope with the increasing energy request during sustained effort or nutrient deprivation, skeletal muscles save plasma glucose or postpone the utilization of muscle glycogen. The inability to shift toward lipid oxidation may underlie the development of insulin resistance [68], whereas an early switch toward lipid-derived energy is observed in SOD1 mice [62].

In contrast to aerobically unfit individuals, ALS mice do show neither signs of insulin resistance nor blunted fat oxidation but the inability to use energy stores in the form of glucose and muscle glycogen and premature shifting toward lipid utilization. It seems as if ALS mice were in a persistent state of starvation and deficiency of energy intake and glycogen storage as primary energy substrate. Intriguingly, age-associated defective mitochondrial function and reduced respiratory coupling efficiency has been observed to affect selectively fast-twitch glycolytic fibers such as gastrocnemius and quadriceps [68].

## 5. Energy Sensor and Fuel Switch: The AMP-Activated Kinase (AMPK) and Hypermetabolism in ALS

Cellular energy status requires to be continuously sensed and adjusted according to the mutable energy needs. The AMPK activation depends on the phosphorylation of threonine 172 that is located within the alpha catalytic subunit of AMPK heterotrimeric protein. Threonine 172 is sensitive to cellular AMP/ATP ratio; thus, a cellular energy deficit or increase in AMP/ATP ratio triggers AMPK phosphorylation and activation. AMPK activation preserves ATP by inhibiting anabolic processes and stimulating catabolic pathways to reinstate ATP-generated cell energy stores [69]. Accordingly, AMPK phosphorylation inhibits glycogen and protein synthesis and stimulates lipid metabolism in skeletal muscle, whereas chronic AMPK activation can promote mitochondrial biogenesis. Evidence for the involvement of AMPK deregulation in ALS pathogenesis has been previously described (see for review [70]).

Within the context of hypermetabolism in ALS and shift in skeletal muscle energy charge, the activity of AMPK becomes of critical importance to regulate anabolic and catabolic pathways to match energy supply and energy expenditure. Muscle contraction is the most effective activator of AMPK, and switching from glycolytic type IIb fibers to oxidative type I or type IIa fibers is the best energy-enhancing strategy during the execution of endurance movement. Thus, AMPK activation during physical exercise promotes type IIb to type IIa fiber transition and chronic treatment with the AMPK mimetic AICAR increases the expression of UCP-3 and peroxisome proliferator-activated receptor-*γ* coactivator-1*α* (PGC-1*α*) [71, 72]. Although type IIb to type IIa fiber transition has not yet been investigated in ALS mice models, increased AMPK phosphorylation has been detected in motor neurons of ALS patients [73] and at spinal cord and motor neuron level in SOD1 mice [74]. AMPK stimulation may worsen disease in female animals [75], whereas its inhibition may improve locomotor function in *Caenorhabditis elegans*-expressing human SOD1 [76]. The activity of AMPK affects also mislocalization of transactive response (TAR) DNA-binding protein-43 (TDP-43) from the nucleus to the cytoplasm in motor neuronal cell line as well as in the spinal cord of ALS patients [77] as reported by a paper from Liu and collaborators. These authors, furthermore, showed that the inhibition of AMPK activity rescues the mislocalization of TDP-43 in cultured cells and delays disease progression in TDP-43 transgenic mice. In spite of the above evidence, latrepirdine- or resveratrol-induced AMPK activation has shown protective effects or delayed symptom onset have been reported in SOD1 mice [78, 79]. However, resveratrol is not selective for AMPK and also activate and increase Sirtuin 1 expression [78]. On the other hand, latrepirdine is a multitarget antihistaminergic drug showing antagonistic activity at *α*-adrenergic and serotonergic receptors and significant proautophagic activity [79]. A possible key to understanding some conflicting data might be considering the role of AMPK in the regulation of autophagy flux and the role played by PGC-1*α* as a biomolecular link between the transcriptional machinery, mitochondrial biogenesis, adaptive thermogenesis, and oxidative metabolism, which can be induced by several stimuli such as physical exercise, cold, and nutrient depletion [80]. Indeed, AMPK promotes oxidative metabolism through PGC-1*α* expression and simultaneous inhibition of mTOR activation and autophagy stimulation. PGC-1*α* expression seems to confer neuroprotection in ALS mice and its upregulation at muscle level to improve muscle function and mitochondrial biogenesis [81, 82]. It is, however, worth mentioning that the skeletal muscle-restricted over expression of PGC-1*α* in ALS mice did not modify survival of this animal model [82] highlighting, once again, the possible involvement of other cellular species to the process that leads to the motor neuron degeneration. Remarkably, PGC-1*α* mRNA expression was found altered with an early decrease of PGC-1*α*-associated mitochondrial function in skeletal muscle of SOD1 mice and ALS patients [83]. Brain downregulation of PGC-1*α* gene *Ppargc1a* in the CNS (i.e., spinal cord and brainstem) but also tissue-restricted peripheral upregulation of *Ppargc1a* has been described in muscle of SOD1 mice and in brown adipocytes from neonate *Fus^ΔNLS/ΔNLS^* homozygous mice [84]. Endurance exercise is a powerful regulator of muscle PGC-1*α* expression and, in turn, the expression of PGC-1*α* is a dynamic regulator of fiber type transition, angiogenesis, muscle metabolism, and insulin sensitivity. PGC-1*α* expression is higher in oxidative slow-twitch type I fibers than in type II glycolytic muscle, and overexpression of PGC-1*α* induces the downregulation of the MHC glycolytic isoforms (MHCIIx and MHCIIb) while it upregulates the oxidative MHC isoforms and oxidative metabolism [85]. Both upregulation of *Ppargc1* gene in skeletal muscle and increased lipid metabolism in fast-twitch type IIb fibers of SOD1 mice [62, 84] support the notion that the energy derived from muscle glycogen might gradually decline favoring an increase of plasma fatty acid oxidation and possibly hypermetabolism in ALS.

## 6. From Lipid Metabolism to Interorgan Communication in ALS: “The Good, the Bad and the Ugly”

Dyslipidemia is well described in ALS patients and severe dyslipidemia such as increased cholesterol or triglycerides levels, and high LDL to HDL ratio is considered protective and associated with a better diagnosis [86, 87]. Support to high caloric enteral nutrition as strategy of dietary intervention for ALS patients has been provided recently [88]. According to a Japan survey, low-fat dietary habits and high carbohydrate intake is associated with higher ALS risk [89]. Exposure to high-fat diet (HFD) can improve survival in SOD1 mice [90] and exposure of TDP-43 mice to HFD significantly delayed AMPK phosphorylation and extended survival, thus supporting the notion that providing high-energy diet can help to rebalance bioenergetic stress in ALS pathophysiology [91]. Moreover, in SOD1 mice, caloric restriction further aggravates motor neuron survival in spinal cord, reduces lifespan, and exacerbates AMPK activation while the opposite is observed after exposure to HFD [91]. Increased fat metabolism and severe loss of body fat depots has been described in mutant mice bearing the TDP-43 deletion [92]. The protective effects exerted by high-energy fat diet discloses the possibility that lipid metabolism might be deranged toward a pathologically enhanced lipolysis. The idea was elegantly corroborated by showing the rapid fall in plasma lipid levels and consequent postprandial hypolipidemia in SOD1 mice [93]. The authors found an increased clearance of triglyceride-rich lipoproteins in lack of alterations of liver lipid metabolism. Notably, the increased expression of genes responsible of lipoprotein clearance at skeletal muscle level such as very low-density lipoprotein receptor provides a robust evidence of muscle liability for the increased lipid utilization. Excessive lipolysis has been found concomitant with the expression of pathological CNS acidosis (e.g., increased CO_2_ levels) in SOD1 mice that aggravates as function of ALS progression [94]. The administration in these mice of a carbonic anhydrase inhibitor to block the conversion of CO_2_ to H_2_O and HCO_3_ and further increase acidosis exacerbates the deterioration of motor function. Consistently, increased acidosis was found associated with reduced fat depots and triglyceride levels and elevated glucagon plasma levels and glycogen accumulation in the spinal cord both in SOD1 mice and in ALS patients [94]. Thus, excessive acidosis and glycogen increase may become significant biomarkers and reveal novel pathways for the investigation of metabolic derangement in ALS.

Interestingly, lipodystrophy has been associated with increased autophagy and loss of alpha motor neurons at spinal cord level [95], which belong to the same class of large motor neuron that mostly innervate fast-twitch glycolytic muscle fibers and therefore are preferentially deranged in ALS. Although is not clear yet whether autophagy dysregulation in ALS stems from increased autophagy process or impairment of autophagy flux, it should be noted that drug-induced autophagy in SOD1 mice did not relieve spinal cord SOD1 aggregates and that increase of autophagy activity exacerbated motor neuron degeneration [96]. Moreover, considering the insufficient nutritional status showed in ALS mice models and ALS patients, it might be worth evaluating the contribution played by caloric loading and its impact on autophagy inhibition. Both alterations of lipid metabolism and the unfavourable prognosis in subjects with lower BMI have encouraged the study of the dysfunction of adipose tissue in ALS patients.

Hence, although not yet investigated, the role of nutrient depletion and caloric restriction as autophagy inducers should be considered, given the state of insufficient nutritional status present in both mice models and ALS patients. Despite altered lipid metabolism in ALS patients and the unfavourable prognosis in subjects with lower BMI, there is information concerning the alterations of adipose tissue in ALS patients. Interestingly, a recent population study has provided evidence for selective hormonal and adipokine alterations in ALS patients and in particular lower ghrelin, glucagon-like peptide-1 (GIP), and pancreatic polypeptide (PP) plasma levels as well as increased metabolic disease-associated adipokines such as interleukin-6 (IL-6), interleukin-8 (IL-8), lipocalin-2 (LCN-2), tumor necrosis factor alpha (TNF*α*), and plasminogen activator inhibitor-1 (PAI-1) [97]. The increase in IL-6 and IL-8 expression is in line with the possible development of insulin (including hepatic) resistance and impaired glucose tolerance reported in ALS patients [65]. Moreover, an increased expression of not only TNF*α*, IL-6, and IL-8 but also LCN-2 and PAI-1 are compatible with different inflammatory processes such as dyslipidemia and neuroinflammatory conditions, activated microglia, neurotoxicity, and defective immune response. Notably, LCN-2 upregulation is relevant in sustained inflammation of adipose tissue and, recently, has also been demonstrated to activate the melanocortin 4 receptor- (MC4R-) dependent pathway and induce anorexigenic effects [98]. Since prolonged exercise can induce IL-6 secretion from skeletal muscle and mediate adaptive responses in adipose tissue (fatty acid oxidation and lipolysis), the IL-6 can be considered involved in the crosstalk between muscle and adipose tissue. In this view, the overexpression of IL-6 in ALS patients corroborates the idea of sustained fatty acid oxidation and lipolysis as well as that slow-twitch oxidative (type I) fibers may be a primary target in the pathophysiology of ALS. The recognized endocrine functions of muscle and adipose tissue has led to the concept of interorgan communication between muscle, fat, and bone which represents an innovative way of thinking about multifactorial and multiorgan diseases.

Bone is not an inert tissue but an endocrine organ involved in energy metabolism [99], and the osteoblast-derived endocrine factor osteocalcin regulates insulin signaling and improves sensitivity and glucose homeostasis [100]. Except for the increase of LCN-2 plasma levels in patients with ALS [97], the role of osteoblast-derived LCN-2 has never been investigated in ALS. However, bone cells are involved in energy metabolism and the fact that LCN-2 suppresses food intake in a leptin-like fashion [98, 99] might provide an attractive link between malnutrition and weight loss in ALS patients with the alteration of LCN-2 levels. Leptin induces thermogenesis via the sympathetic nervous system (SNS) and innervation of interscapular brown adipose tissue and also by activating leptin-responsive thyrotropin-releasing hormone (TRH) neurons within the hypothalamus [97]. In line with this hypothesis, G93A SOD1 mice showed an improvement of energy homeostasis and a slow disease progression when placed in a leptin-deficient background [101].

At variance with the idea of a possible relationship between LCN-2 upregulation and the anorexigenic effects induced by the overactivity of the melanocortin 4 receptor- (MC4R-) pathway [97, 98], pioglitazone treatment failed to stimulate an increase of body weight in ALS patients and in SOD1 mice [102], a lack of effect described by the hypothesis of defective melanocortin system and downregulation of proopiomelanocortin (POMC) neurons in ALS. As other thiazolinediones (TZDs), pioglitazone is a peroxisome proliferator-activated receptor gamma (PPAR*γ*) agonist that stimulates food intake and body weight gain by downregulating the anorexigenic *α*-melanocyte stimulating hormone (MSH) via the reduced synthesis of its precursor POMC. At first sight, this is problematic to reconcile with reduced energy intake and decreased body weight observed in ALS, although an increase of food (namely lipid) intake has been described in presymptomatic ALS patients that also exhibited lower premorbid BMI [103].

Overall, data depicting a possible melanocortin malfunctioning in ALS let us point out again that hypermetabolism might represent a counter-regulatory response to excessive energy wasting. Within this context, energy-dense food and/or high palatability and high rewarding food might be a solid option to reinstate energy accumulation in the face of reduced adipose depots. In ALS, patients have reported changes in eating behavior and macronutrient intake that develop parallel with the deterioration of cognitive profile [104] as well as a positive correlation between severity of eating behavior changes and survival. The parallel between eating and cognitive changes supports the notion that brain regulation of eating behavior, and in particular the hypothalamic pathways controlling eating homeostasis, are of critical relevance in ALS pathogenesis as further demonstrated by the presence of pathologic TDP-43 inclusions in the lateral hypothalamus of ALS patients with associated decrease of BMI [105].

With reference to the failure of pioglitazone to stimulate body weight and adiposity in ALS, there might be other reasons to consider. Indeed, pioglitazone-induced increase of body weight and food intake is also associated with the serum increase of adiponectin and activation (i.e., phosphorylation) of hypothalamic AMPK [106]. Accordingly, selective hypothalamic inhibition of AMPK and downregulation of hypothalamic adiponectin receptor 1 (AdipoR1) offset pioglitazone-induced adiposity, food intake, and increased energy expenditure [106]. Hence, it would be interesting to assess whether AMPK expression is altered (putatively reduced) in SOD1 mice and so verify the hypothesis that hypothalamic downregulation of AMPK-mediated signaling might contribute to explain reduced appetite and energy intake in ALS. The central orexigenic effects of ghrelin are mediated via AMPK activation [107] that, upon its phosphorylation, stimulates fatty acid oxidation and the downstream activation of agouti-related peptide/neuropeptide Y (AGRP/NPY) orexigenic neurons. However, ghrelin peripheral levels are decreased in ALS patients [93] and, surprisingly, peripheral ghrelin has been demonstrated to mediate the activation of antiatrophic pathways in skeletal muscle and reduce muscle wasting [108]. Like ghrelin, adiponectin activates hypothalamic AMPK via the AdipoR1 and stimulates fatty acid oxidation and food intake, whereas the lack of hypothalamic adiponectin reduces AMPK activation together with a decrease of food consumption and an increase of energy expenditure [109]. It is worth reminding that pioglitazone fails to induce food intake when AMPK and AdipoR1 are downregulated [106] and that reduced hypothalamic adiponectin- and AMPK-mediated signaling may promote energy expenditure. Interestingly, derangement of energy homeostasis is reported also in frontotemporal dementia (FTD) and the specific clinical continuum between ALS and FTD has been critically highlighted [110]. Comorbidity of ALS and FTD is not infrequent, and concomitant diagnosis of FTD is recognized in at least 15% of ALS patients [110]. Alterations in eating behavior are described in FTD patients and in some clinical variants such as the behavioral variant (bvFTD) and the variant of fluent language presentation or semantic variant of primary progressive aphasia (svPPA) [111]. As reported, these subjects display abnormal appetite with increased energy intake in the form of higher ingestion of sugar and carbohydrate and a general higher preference for sweet food. Abnormalities of eating behavior in FTD have been associated with morphological alterations of the insula, striatum, orbitofrontal cortex, and posterior hypothalamus [110]. Hence, the two major clusters of metabolic symptoms in ALS and FTD appear to show either commonalities or disparities depending on the clinical aspects of the metabolic spectrum analyzed. Indeed, in ALS, the disruption of eating behavior not only can assume the form of dysphagia, difficulty in meal swallowing, and weight loss but also can increase saturated fat intake [104], while an overall increase of caloric intake and even hyperphagia are described in FTD [110]. By contrast, defective energy homeostasis appears to assume a similar profile particularly for the comparison between ALS patients and patients affected by bvFTD. As in ALS clinical spectrum, an increase of resting energy expenditure, basal metabolic rate, and resting heart rate have been found in patients with bvFTD [112]. A further elucidation of the mechanisms underlying eating abnormalities and energy homeostasis in ALS and FTD may help to better disclose the association between neurodegeneration and metabolic derangement.

Hence, to further understand the mechanisms underlying hypermetabolism in ALS, we need to better focus on the hypothalamic signaling pathways involved in the regulation of energy expenditure. In addition to the above recalled mechanisms responsible for leptin-induced thermogenesis (e.g., [101]), leptin induces thermogenesis in skeletal muscle [113], and a recent paper has disclosed the involvement of a new population of leptin-responsive prolactin-releasing neurons in the dorsomedial hypothalamus [114]. Notably, hypothalamic nuclei (i.e., ventromedial (VMH)) controls not only brown adipose tissue- (BAT-) induced thermogenesis but also glucose metabolism in skeletal muscle, mainly in type I oxidative fibers [115]. Recently, the direct activation of VMH melanocortin receptors demonstrates to increase energy expenditure and lipid oxidation via the stimulation of SNS-mediated skeletal muscle temperature [116].

**Figure 1:**
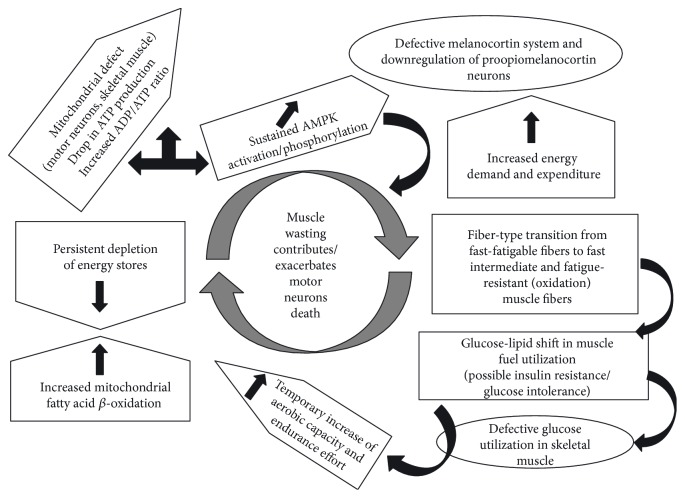
The viciouc loop of hypermetabolism in ALS.

In concluding our survey, we believe that a deep understanding of the possible origin of hypermetabolism in ALS (see Figure 1) might offer a novel viewpoint to look at the elusive matter of ALS aetiology. Lately, several “axes” have been disclosed such as brain-muscle axis or the adipose-brain axis. Thus, we believe that a brain-muscle-fat signaling axis is of special interest for the investigation of the hidden mechanisms underlying hypermetabolism in ALS. The interorgan communication, including the role of bone metabolism, is still at its dawn, and nevertheless, its impact towards a better understanding of ALS pathogenesis should not be underestimated.
